# Supplementary figures and images for: Transcriptome analysis revealed gene regulatory network involved in PEG-induced drought stress in Tartary buckwheat (Fagopyrum Tararicum)
Source: PeerJ. 2021 Mar 31;9:e11136. doi: 10.7717/peerj.11136 (PMC8019315; doi:10.7717/peerj.11136)

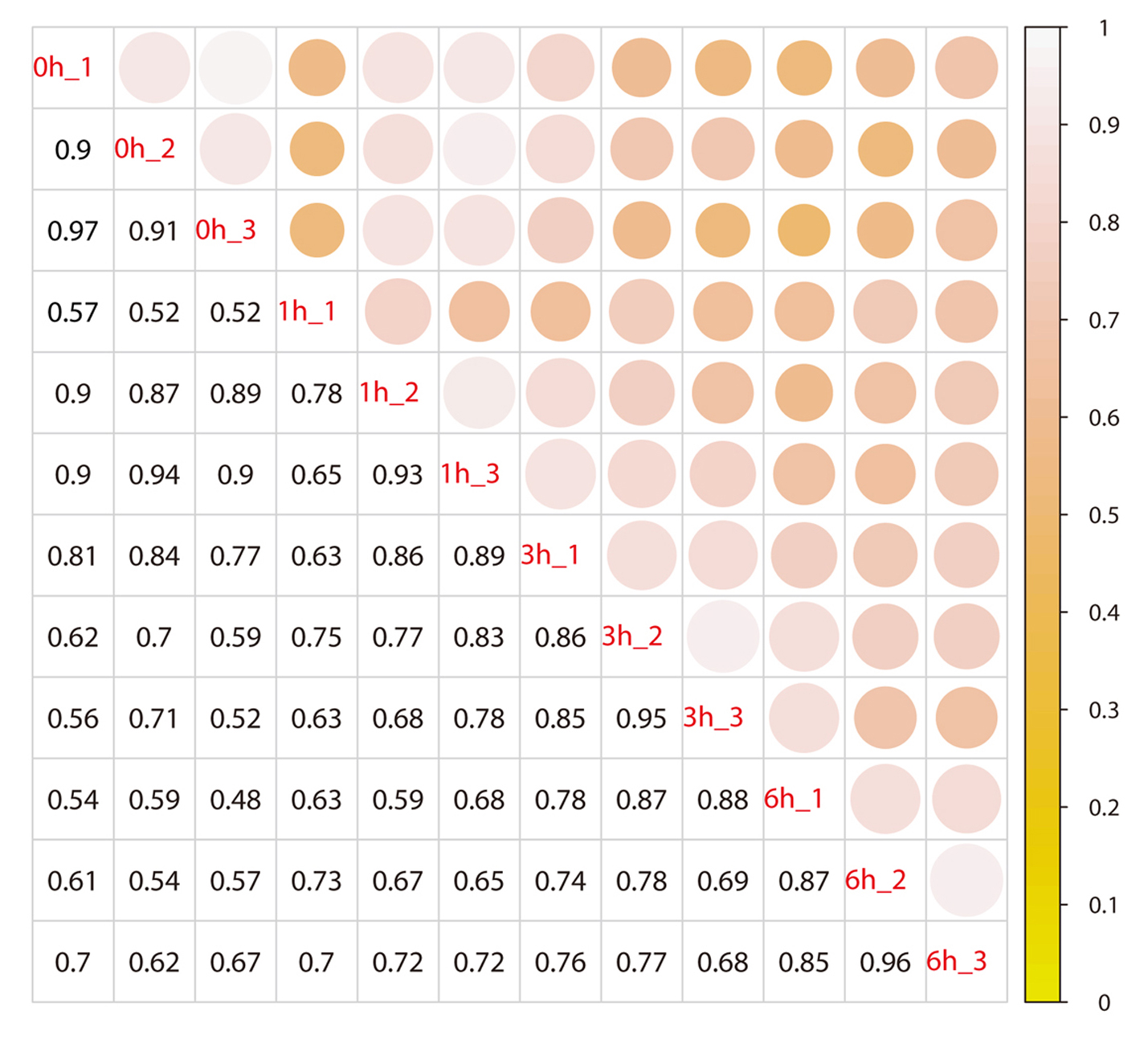

Supplement: Figure S1 [file peerj-09-11136-s001.jpg]

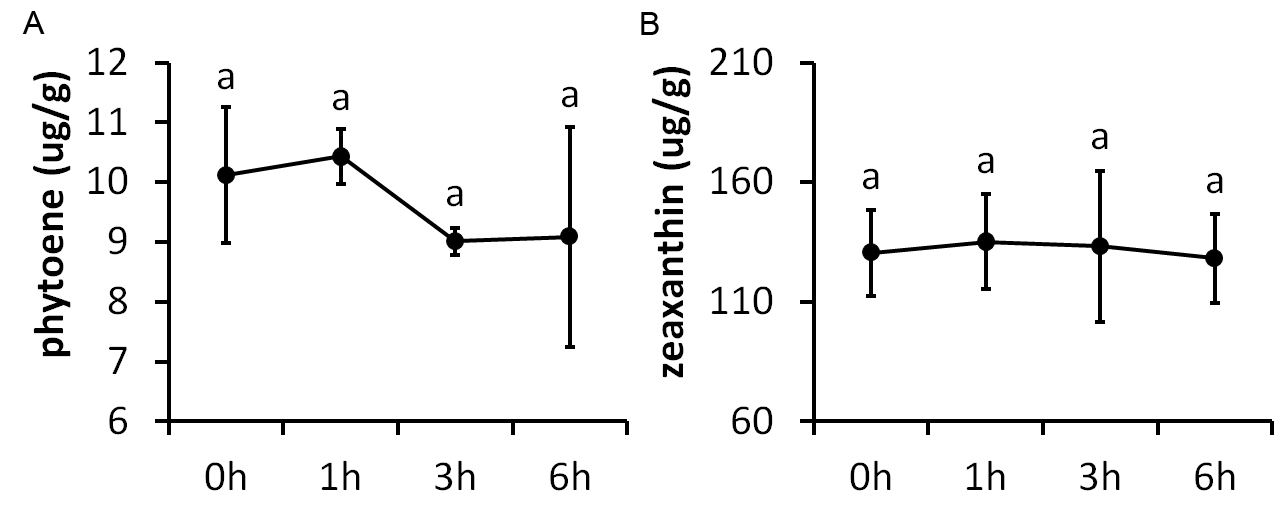

Supplement: Figure S2 [file peerj-09-11136-s002.jpg]
